# Supplementary material for: Unravelling the French National Fungal Database: Geography, Temporality, Taxonomy and Ecology of the Recorded Diversity
Source: J Fungi (Basel). 2022 Aug 31;8(9):926. doi: 10.3390/jof8090926 (PMC9504494; doi:10.3390/jof8090926)
Supplement: Supplementary file 1 [file jof-08-00926-s001.zip › jof-1857721-supplementary.pdf]

Acknowledgments

This article is dedicated to the memory of Christian Lechat, we founded AscoFrance. The work was co-funded by AdoniF and the Observatoire de Recherche Méditerranéen de l'Environnement (OREME). We thank the Ministère de la Transition Ecologique and Julien Touroult and Pascal Dupont from the UMS PatriNat for financial support to AdoniF for the project FongiFrance. We're grateful to all contributors to FongiBase database, especially Jean-Claude Malaval, Jean-Pierre Dubus and Jean-Paul Maurice, the members of AMO (Association Mycologique de l'ouest), SMRY (Société Mycologique de la Roche sur Yon), SMF (Société Mycologique de France), FMBDS (Fédération mycologique et botanique Dauphiné-Savoie, MycoflAURA program managed by Nicolas Van Vooren), SMNF (Société Mycologique du Nord de la France), RNF (Réserves Naturelles de France), MNHN (Muséum National d'Histoire Naturelle), the University of Lille, the Conservatoire Botanique National Franche-Comté (CBNFC-ORI) and its mycological expert Daniel Sugny, AscoFrance and AdoniF.. We are endebted to Régis Courtecuisse and Alain Delannoy for having provided all the data collected during the Programme National D'Inventaire et de Cartographie des Mycota français. We are particularly grateful to Alexia Vaillé and Berangère Baldit, from the administrative support of OREME, for their technical assistance. We thank Anders Dahlberg and Gregory Mueller for enlightening discussion and advices at the early stage of the project

Conflicts of Interest

The authors whose names are listed immediately below certify that they have NO affiliations with or involvement in any organization or entity with any financial interest (such as honoraria; educational grants; participation in speakers' bureaus; membership, employment, consultancies, stock ownership, or other equity interest; and expert testimony or

patentlicensing arrangements), or nonfinancial interest (such as personal or professional relationships, affiliations, knowledge or beliefs) in the subject matter or materials discussed in this manuscript.

Author names: Montan Gautier a, Pierre-Arthur Moreau b, Béatrice Boury b, Franck Richard.

Supplementary Material

Figure legend

Figure S1 Attribution of four ecoregions in France by departments. The dotted line indicates the geographical limits of ecoregions without department administrative borders. The departments 75 (Paris), 92 (Hauts-de-Seine), 93 (Seine-Saint-Denis), and 94 (Val-de-Marne) are represented by a red star and they are assigned to the Atlantic ecoregion.

Figure S2. Taxon accumulation curve for the four clusters of records in France. The color of curves indicates the corresponding ecoregion, with brown, blue and green for montane, Atlantic and continental domains, respectively.

Figure S3. Distribution of taxa based on their occurrence in the database, ordered by observation classes using **A.** multiples of 100 records **B.** for taxa with less than 100 records (red bar in panel A), multiples of 10 records and **C.** for taxa with less than 10 records (red bar in panel B) detailed distribution per each frequency.

Figure S4. Temporal dynamics of fungal recording during the period [1800-2022[in four clusters of recording activity. The Northern cluster includes the departments of Nord, Oise, Nord-Pas-de-Calais and Seine-Maritime. The Western cluster includes the departments of Charentes, Loire-Atlantic, Mayenne, Vendée and Vienne. The Eastern cluster includes the departments of Doubs, Jura, Haute-Saône and Territoire de Belfort. The Alpine cluster includes the departments of Isère, Loire, Rhône, Savoie and Haute-Savoie.

Supplementary Tables

Table S1: Identity of the 26 contributors of FongiBase with the number of deposited fungal observations and the number of one observation taxa (singletons).

**Contributor**	**Number of records**	**Percentage of records**	**Number of singletons**	**Proportion of singletons (in %)**
ABMARS	7262	0.7	3	0.04
ADONIF	120394	11.9	372	0.31
AMO	47515	4.7	100	0.21
AscoFrance	2385	0.2	239	10.02
Amblard BD.	1	0.00009	0	0
CEMA	48	0.005	0	0
Lécuri C., Moreau PA.	123	0.01	1	0.81
CBNFC-ORI	177838	17.6	37	0.02
Faculté de pharmacie de Rennes	168	0.02	0	0
FMBDS	180358	17.9	624	0.35
GMN	1765	0.2	3	0.17
Le Pleurote Sinois	5967	0.6	0	0
MNHN	155758	15.4	173	0.11
ONF	314	0.03	0	0
Aleksandrowicz P., Chabrerie O., Clowez P.	1	0.00009	0	0
RNF	136257	13.5	84	0.06
SMCTO	7670	0.7	0	0
SMF	15061	1.5	178	1.18
SMNF	112917	11.2	329	0.29
SMP	16	0.002	0	0
SLNP	5570	0.6	7	0.13
SMRY	4272	0.4	97	2.27
SMM	1449	0.1	0	0
SMR	752	0.1	3	0.40
ULCO	33	0.0	2	6.06
UL	42001	4.2	97	0.23

Table S2: Detail of the number of deposited fungal records in FongiBase in relation to their natural habitat of collecting according to CORINE Biotopes catalog.

CORINE biotope code	CORINE biotope division 1	CORINE biotope division 2	Number of records	Percentage of records
2	Non-marine waters	Non-marine waters unclassified	104	0.08
3	Scrub and grasslands	Scrub and grassland unclassified	1488	1.21
4	Forests	Forests unclassified	2154	1.75
5	Bogs and marshes	Bogs and marsh unclassified	544	0.44
6	Rocky habitats	Inlands rocks, screes and sands unclassified	167	0.14
8	Agricultural land and highly artificial landscapes	Agricultural land and highly artificial landscapes unclassified	224	0.18
16	Coastal and halophilic habitats	Coastal sand-dunes and sand beaches	2474	2.01
22	Non-marine waters	Standing fresh water	139	0.11
31	Scrub and grasslands	Heath and scrub	1699	1.38
32	Scrub and grasslands	Sclerophyllous scrub	109	0.09
34	Scrub and grasslands	Dry calcareous grasslands and steppes	3687	3.00
35	Scrub and grasslands	Dry siliceous grasslands	424	0.35
36	Scrub and grasslands	Alpine and subalpine grasslands	3406	2.77
37	Scrub and grasslands	Humid grasslands and tall herb communities	860	0.70
38	Scrub and grasslands	Mesophile grassland	754	0.61
41	Forests	Broad-leaved deciduous forests	25289	20.58
42	Forests	Coniferous woodland	18008	14.66
43	Forests	Mixed woodland	44525	36.24
44	Forests	Alluvial and very wet forests and brush	3543	2.88
45	Forests	Broad-leaved evergreen woodland	179	0.15
51	Bogs and marshes	Raised bogs	335	0.27
53	Bogs and marshes	Water-fringe vegetation	125	0.10
54	Bogs and marshes	Fens, transition mires and springs	4877	3.97
62	Rocky habitats	Inland cliffs and exposed rocks	41	0.03
81	Agricultural land and highly artificial landscapes	Improved grasslands	1834	1.49
82	Agricultural land and highly artificial landscapes	Crops	144	0.12
83	Agricultural land and highly artificial landscapes	Orchards, groves and tree plantat	250	0.20
84	Agricultural land and highly artificial landscapes	Tree lines, hedges, small woods, bocage, parkland dehesa	129	0.10
85	Agricultural land and highly artificial landscapes	Urban parks and large gardens	5335	4.34

86	Agricultural land and highly artificial landscapes	Towns, villages, industrial sites	18	0.01

Table S3: List of French department and associated numbers of records deposited in FongiBase, area and density of observations.

INSEE number	Name of department	Number of records	Area	Record density	Percentage deviation from national observations	Percentage deviation from national density
1	Ain	13,192	5,762	2.29	23.33	23.00
2	Aisne	13,795	7,362	1.87	28.96	0.67
3	Allier	9,694	7,340	1.32	-9.38	-29.05
4	Alpes-de-Haute-Provence	1,814	6,925	0.26	-83.04	-85.93
5	Hautes-Alpes	1,444	5,549	0.26	-86.50	-86.02
6	Alpes-Maritimes	3,834	4,299	0.89	-64.16	-52.09
7	Ardèche	5,054	5,529	0.91	-52.75	-50.89
8	Ardennes	1,649	5,229	0.32	-84.58	-83.06
9	Ariège	7,074	4,890	1.45	-33.87	-22.28
10	Aube	19,336	6,004	3.22	80.76	73.02
11	Aude	1,362	6,139	0.22	-87.27	-88.08
12	Aveyron	3,217	8,735	0.37	-69.93	-80.21
13	Bouches-du-Rhône	1,196	5,088	0.24	-88.82	-87.37
14	Calvados	4,583	5,548	0.83	-57.16	-55.62
15	Cantal	8,221	5,726	1.44	-23.15	-22.87
16	Charente	35,341	5,956	5.93	230.39	218.78
17	Charente-Maritime	12,140	6,864	1.77	13.49	-4.98
18	Cher	1,864	7,235	0.26	-82.57	-86.16
19	Corrèze	768	5,857	0.13	-92.82	-92.96
21	Côte-d'Or	5,727	8,763	0.65	-46.46	-64.89
22	Côtes-d'Armor	13,690	6,878	1.99	27.98	6.93
23	Creuse	1,632	5,565	0.29	-84.74	-84.24
24	Dordogne	4,621	9,060	0.51	-56.80	-72.60
25	Doubs	75,227	5,233	14.38	603.26	672.32
26	Drôme	7,513	6,530	1.15	-29.76	-38.19
27	Eure	9,749	6,040	1.61	-8.86	-13.28
28	Eure	491	5,880	0.08	-95.41	-95.51
29	Finistère	1,580	6,733	0.23	-85.23	-87.39
2A	Corse-du-Sud	871	4,014	0.22	-91.86	-88.34
2B	Haute-Corse	1,029	4,066	0.25	-90.38	-86.40
30	Gard	2,761	5,853	0.47	-74.19	-74.66
31	Haute-Garonne	3,796	6,309	0.60	-64.51	-67.68
32	Gers	358	6,256	0.06	-96.65	-96.93
33	Gironde	1,399	9,976	0.14	-86.92	-92.47

34	Hérault	7,990	6,101	1.31	-25.31	-29.64
35	Ille-et-Vilaine	1,556	6,775	0.23	-85.45	-87.66
36	Indre	109	6,791	0.02	-98.98	-99.14
37	Indre-et-Loire	1,749	6,127	0.29	-83.65	-84.66
38	Isère	50,018	7,432	6.73	367.60	261.57
39	Jura	70,851	4,999	14.17	562.35	661.44
40	Landes	2,432	9,243	0.26	-77.26	-85.86
41	Loir-et-Cher	4,271	6,343	0.67	-60.07	-63.83
42	Loire	15,732	4,781	3.29	47.07	76.78
43	Haute-Loire	8,417	4,977	1.69	-21.31	-9.14
44	Loire-Atlantique	25,971	6,809	3.81	142.79	104.92
45	Loiret	4,977	6,775	0.73	-53.47	-60.53
46	Lot	1,252	5,217	0.24	-88.30	-87.11
47	Lot-et-Garonne	125	5,361	0.02	-98.83	-98.75
48	Lozère	853	5,167	0.17	-92.03	-91.13
49	Maine-et-Loire	8,545	7,172	1.19	-20.12	-35.99
50	Manche	952	5,938	0.16	-91.10	-91.39
51	Marne	1,604	8,169	0.20	-85.00	-89.45
52	Haute-Marne	2,162	6,211	0.35	-79.79	-81.30
53	Mayenne	22,810	5,175	4.41	113.24	136.80
54	Meurthe-et-Moselle	3,746	5,246	0.71	-64.98	-61.64
55	Meuse	2,431	6,211	0.39	-77.27	-78.97
56	Morbihan	2,254	6,823	0.33	-78.93	-82.25
57	Moselle	3,600	6,216	0.58	-66.35	-68.89
58	Nièvre	2,255	6,817	0.33	-78.92	-82.23
59	Nord	58,089	5,743	10.11	443.05	443.41
60	Oise	19,158	5,860	3.27	79.10	75.64
61	Orne	9,878	6,103	1.62	-7.66	-13.04
62	Pas-de-Calais	78,199	6,671	11.72	631.05	529.77
63	Puy-de-Dôme	10,565	7,970	1.33	-1.23	-28.78
64	Pyrénées-Atlantiques	2,575	7,645	0.34	-75.93	-81.90
65	Hautes-Pyrénées	6,846	4,464	1.53	-36.00	-17.61
66	Pyrénées-Orientales	12,698	4,116	3.09	18.71	65.74
67	Bas-Rhin	3,138	4,755	0.66	-70.66	-64.55
68	Haut-Rhin	6,153	3,525	1.75	-42.48	-6.22
69	Rhône	15,204	3,249	4.68	42.14	151.41
70	Haute-Saône	34,777	5,360	6.49	225.11	248.58
71	Saône-et-Loire	1,285	8,575	0.15	-87.99	-91.95
72	Sarthe	993	6,206	0.16	-90.72	-91.40
73	Savoie	34,986	6,028	5.80	227.07	211.81
74	Haute-Savoie	40,103	4,388	9.14	274.90	391.00
75	Paris	818	105	7.79	-92.35	318.54
76	Seine-Maritime	23,710	6,278	3.78	121.65	102.90
77	Seine-et-Marne	12,827	5,915	2.17	19.91	16.50

78	Yvelines	4,399	2,284	1.93	-58.88	3.47
79	Deux-Sèvres	7,563	5,999	1.26	-29.30	-32.27
80	Somme	10,580	6,170	1.71	-1.09	-7.88
81	Tarn	720	5,758	0.13	-93.27	-93.28
82	Tarn-et-Garonne	369	3,718	0.10	-96.55	-94.67
83	Var	2,144	5,973	0.36	-79.96	-80.72
84	Vaucluse	1,448	3,567	0.41	-86.46	-78.19
85	Vendée	26,674	6,720	3.97	149.36	113.25
86	Vienne	48,874	6,990	6.99	356.90	275.64
87	Haute-Vienne	909	5,520	0.16	-91.50	-91.15
88	Vosges	5,653	5,874	0.96	-47.15	-48.30
89	Yonne	2,693	7,427	0.36	-74.82	-80.52
90	Territoire de Belfort	11,433	609	18.77	6.88	908.59
91	Essonne	6,697	1,804	3.71	-37.39	99.44
92	Hauts-de-Seine	497	176	2.82	-95.35	51.71
93	Seine-Saint-Denis	221	236	0.94	-97.93	-49.69
94	Val-de-Marne	362	245	1.48	-96.62	-20.62
95	Val-d'Oise	976	1,246	0.78	-90.88	-57.92

Table S4: List of the 25 most recorded taxa in Fongibase, including their lineage and trophic guild of belonging, and the corresponding number of records.

Species	Phylum	Ecological guilds	Number of records
Hypholoma fasciculare	Basidiomycota	Plant saprotroph	7,091
Amanita rubescens	Basidiomycota	Mutualist	6,413
Trametes versicolor	Basidiomycota	Wood saprotroph	5,915
Amanita citrina	Basidiomycota	Mutualist	5,716
Stereum hirsutum	Basidiomycota	Wood saprotroph	5,495
Russula cyanoxantha	Basidiomycota	Mutualist	5,266
Pluteus cervinus	Basidiomycota	Wood saprotroph	4,583
Mycena pura	Basidiomycota	Plant saprotroph	4,496
Laccaria amethystea	Basidiomycota	Mutualist	4,500
Lycoperdon perlatum	Basidiomycota	Plant saprotroph	4,475
Mycena galericulata	Basidiomycota	Wood saprotroph	4,125
Rhodocollybia butyracea	Basidiomycota	Plant saprotroph	4,121
Fomes fomentarius	Basidiomycota	Wood saprotroph	4,031
Amanita muscaria	Basidiomycota	Mutualist	4,021
Inocybe geophylla	Basidiomycota	Mutualist	3,890
Megacollybia platyphylla	Basidiomycota	Plant saprotroph	3,890
Paxillus involutus	Basidiomycota	Mutualist	3,749
Collybia dryophila	Basidiomycota	Plant saprotroph	3,716
Infundibulicybe gibba	Basidiomycota	Plant saprotroph	3,690
Armillaria mellea	Basidiomycota	Plant saprotroph	3,576
Hymenopellis radicata	Basidiomycota	Plant saprotroph	3,501
Russula ochroleuca	Basidiomycota	Mutualist	3,483
Xylaria hypoxylon	Ascomycota	Wood saprotroph	3,461
Fomitopsis betulina	Basidiomycota	Wood saprotroph	3,468
Clitocybe nebularis	Basidiomycota	Plant saprotroph	3,437

Table S5: Detail of the number of deposited fungal records in FongiBase in relation to their belonging to ecological guilds according to FunGuild.

Ecological guilds	Number of records	Percentage of records
Animal parasite	753	0.08
Animal saprotroph	51	0.01
Bryotrophic	5642	0.61
Carbonicolous	294	0.03
Dung saprotroph	6550	0.71
Endophyte	2897	0.32
Epiphyte	53	0.01
Fungal parasite	8804	0.96
Mutualist	409643	44.64
Plant parasite	26631	2.90
Plant saprotroph	289921	31.59
Wood saprotroph	166402	18.13

Figure S1

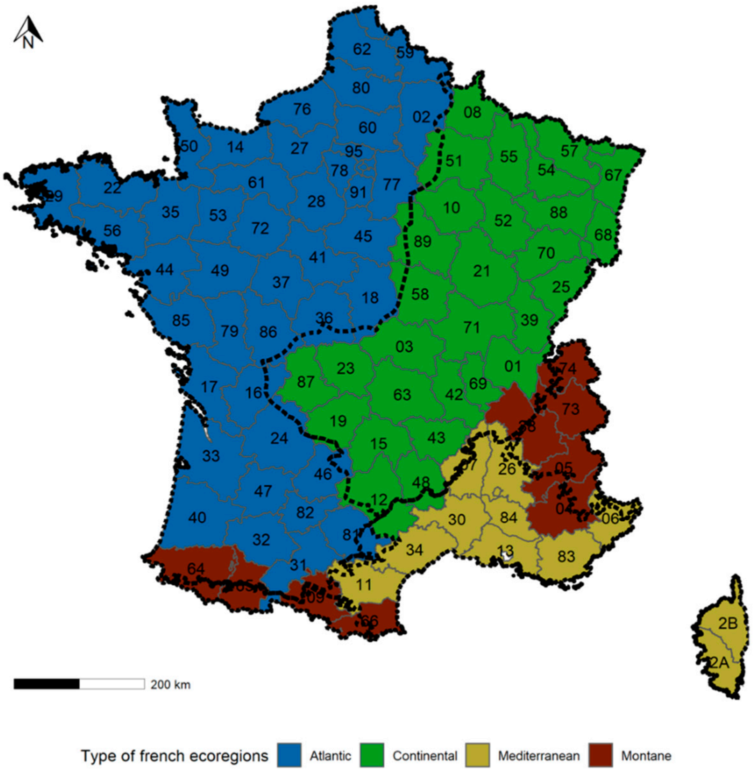
N
200 km
Type of french ecoregions
Atlantic
Continental
Mediterranean
Montane

Figure S2

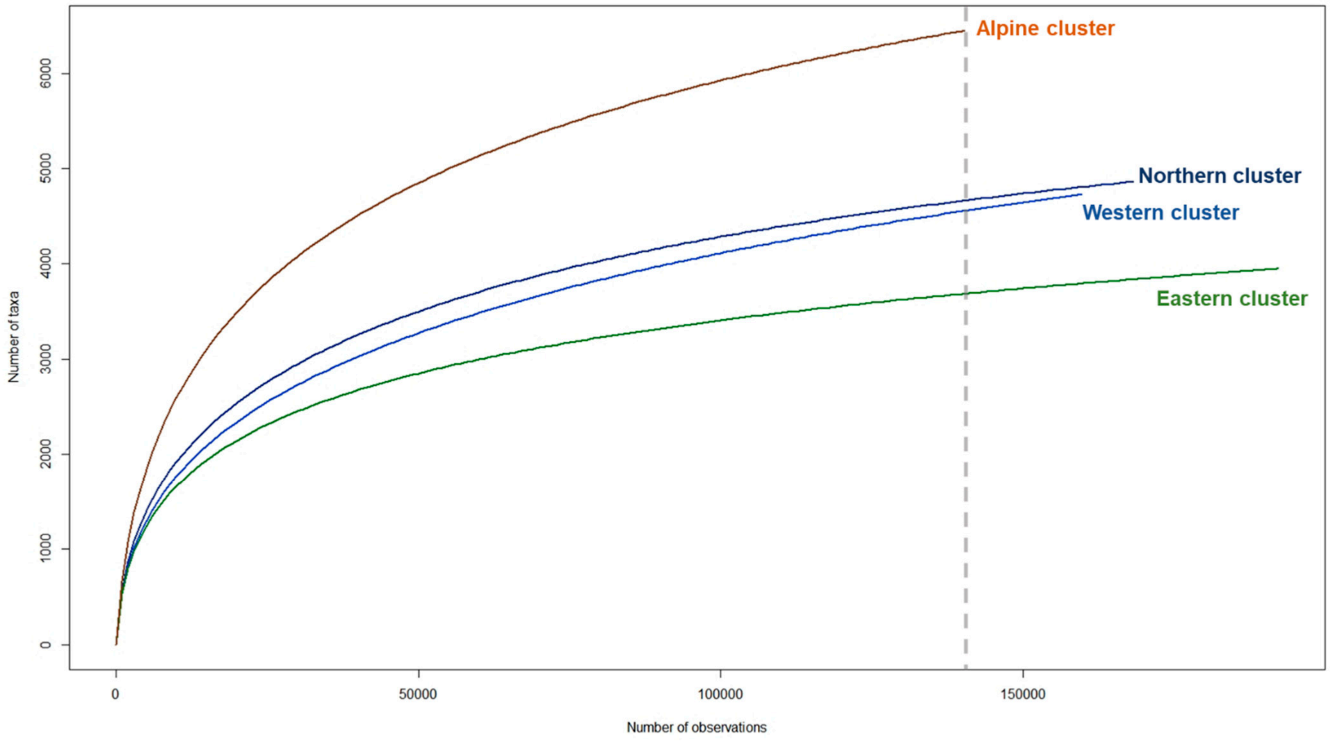

Alpine cluster
Northern cluster
Western cluster
Eastern cluster
Number of taxa
0
1000
2000
3000
4000
5000
6000
0
50000
100000
150000
Number of observations

Figure S3

A **B** **C**

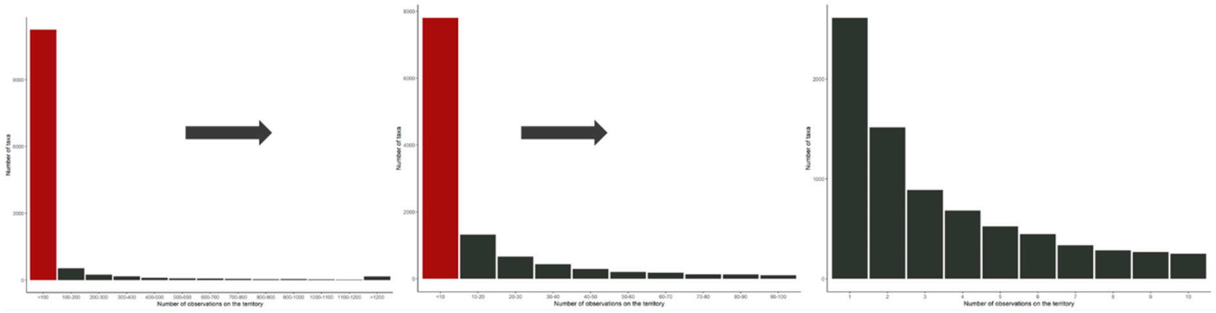

Figure S3. Distribution of taxa based on their occurrence in the database, ordered by observation classes using **A.** multiples of 100 records **B.** for taxa with less than 100 records (red bar in panel A), multiples of 10 records and **C.** for taxa with less than 10 records (red bar in panel B) detailed distribution per each frequency.

Figure S4

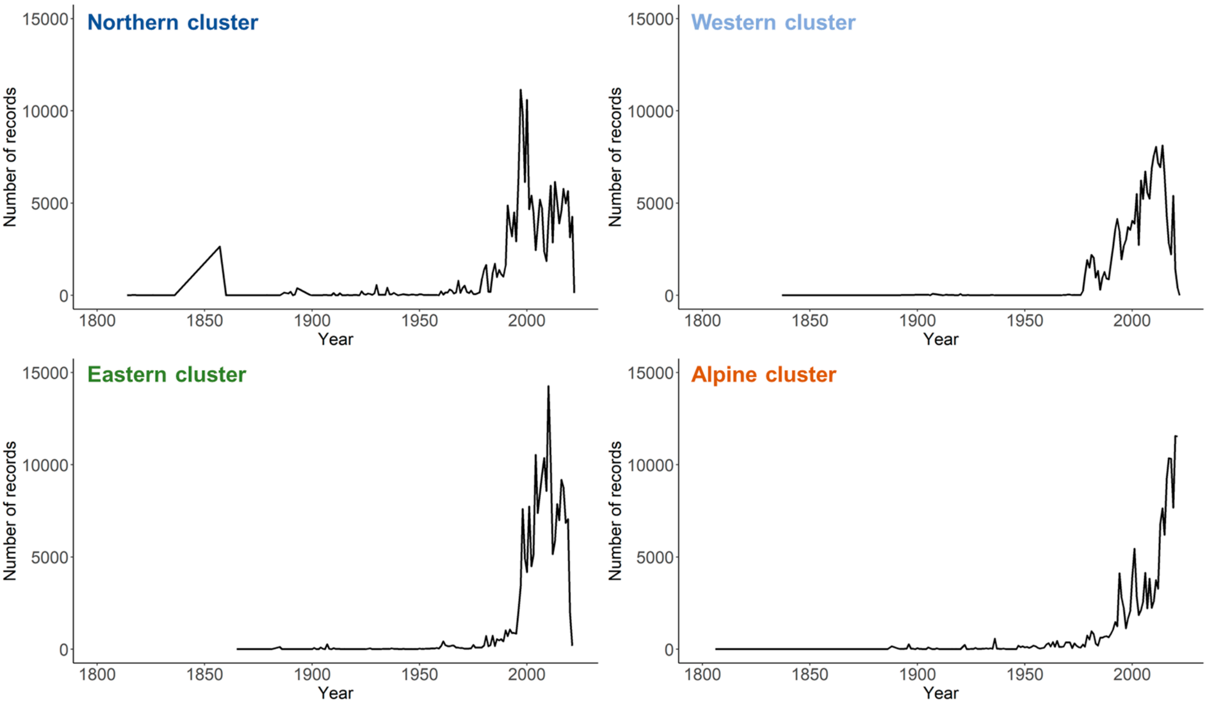

Northern cluster
Western cluster
Eastern cluster
Alpine cluster
Number of records
15000
10000
5000
0
Year
1800
1850
1900
1950
2000
